# Autoimmune Limbic Encephalitis Associated with Type 1 Diabetes Mellitus

**DOI:** 10.4274/jcrpe.3818

**Published:** 2017-12-15

**Authors:** Onur Akın, Aylin Kılınç Uğurlu, Emine Demet Akbaş, Esra Döğer, Yılmaz Akbaş, Aysun Bideci, Özge Yüce, Kıvılcım Gücüyener, Mahmut Orhun Çamurdan, Neşe Karabacak, Peyami Cinaz

**Affiliations:** 1 Gazi University Faculty of Medicine, Department of Pediatric Endocrinology, Ankara, Turkey; 2 Gazi University Faculty of Medicine, Department of Pediatric Neurology, Ankara, Turkey; 3 Gazi University Faculty of Medicine, Department of Nuclear Medicine, Ankara, Turkey

**Keywords:** Limbic encephalitis, diabetes, anti-glutamic acid decarboxylase

## To The Editor,

Limbic encephalitis (LE) is an autoimmune, neurological disorder characterized by confusion, memory disturbance, and seizures. An association between type 1 diabetes mellitus (T1D) and other autoimmune disorders is well-known. However, the co-occurrence of T1D and LE is very rare.

A 16-year-old boy was admitted to our emergency department with confusion and headache. Electroencephalography revealed temporal slowing, cerebral magnetic resonance imaging demonstrated hyperintense signal of the right mesiotemporal lobe, and positron emission tomography demonstrated increased activity in the right temporal lobe. Blood glutamic acid decarboxylase antibody (anti-GAD) level was 2114 IU/mL (0–10) and the cerebrospinal fluid anti-GAD level was 4.07 nmol/L (<0.02). These findings led to a consideration of autoimmune LE as a possible diagnosis. Pulse methylprednisolone was administered over five days. After steroid treatment, symptoms improved, but hyperglycemia occurred on the third day of treatment. Glycemia level reached 502 mg/dL. Concurrent insulin level was 42 µIU/mL. Hyperglycemia improved after cessation of steroid treatment. Glycated hemoglobin was 5.6%. The possibility of a steroid-induced hyperglycemia was considered. Six months later, the patient was readmitted with dyspnea and abdominal pain. The family reported occurrence of polyuria and polydipsia during the previous two months. Blood anti-GAD level was >2000 IU/mL. The patient was diagnosed to have T1D. With treatment, the ketoacidosis improved in 10 h. After being educated for diabetes, the patient was discharged. Two months later, he presented with a headache and confusion again. Intravenous immunoglobulin (IVIG) 1 g/kg/d for two days every month was administered. Neurological symptoms improved and the daily insulin dose was decreased.

GAD catalyzes the production of γ-aminobutyric acid which is the most important inhibitory neurotransmitter. Especially GAD65 is highly expressed in the central nervous system (1). It is also a target antigen in T1D (2). It was reported that the patients with high values of anti-GAD (>2000 IU/mL) encountered neurological disorders (3). A few cases have been reported in which T1D and LE were associated with a high titer of anti-GAD (4). In all these cases, the patients were diagnosed with T1D prior to development of encephalitis symptoms. In contrast, our patient was diagnosed with T1D six months after LE. The diabetes developed during steroid therapy for encephalitis and the patient was initially considered as having a steroid-induced diabetes. The effect of glucocorticoids on glucose metabolism is the result of both beta cell dysfunction and insulin resistance (5). The findings in our patient (42 µIU/mL insulin concurrent with 502 mg/dL glycemia) can be considered as a relative insulinopenia. It can be speculated that in this patient, the pathogenesis of the diabetic state could be a combination of steroid impact and impairment of beta cells due to anti-GAD antibodies during the beginning stages of T1D. We also observed that IVIG administration decreased the need for the average insulin dose. However, it is difficult to distinguish whether the decrease was due to the impact of IVIG or to a honeymoon phase.

In conclusion, there is a possible association between T1D and autoimmune neurologic disorders due to anti-GAD. Close follow-up is important for diabetic patients with anti-GAD to detect neurological deterioration. In addition, patients encountering GAD65-related neurological disorders should be followed carefully for T1D.
